# Experimental and Computational Structural Studies of 2,3,5-Trisubstituted and 1,2,3,5-Tetrasubstituted Indoles as Non-Competitive Antagonists of GluK1/GluK2 Receptors

**DOI:** 10.3390/molecules27082479

**Published:** 2022-04-12

**Authors:** Agata Bartyzel, Agnieszka A. Kaczor, Ghodrat Mahmoudi, Ardavan Masoudiasl, Tomasz M. Wróbel, Monika Pitucha, Dariusz Matosiuk

**Affiliations:** 1 Department of General and Coordination Chemistry and Crystallography, Institute of Chemical Sciences, Faculty of Chemistry, Maria Curie-Skłodowska University in Lublin, M. Curie-Skłodowskiej Sq. 2, PL-20031 Lublin, Poland; 2 Pharmaceutical Substances with Computer Modeling Laboratory, Department of Synthesis and Chemical Technology, Faculty of Pharmacy, Medical University of Lublin, 4A Chodźki St., PL-20093 Lublin, Poland; tomasz.wrobel@umlub.pl (T.M.W.); dariusz.matosiuk@umlub.pl (D.M.); 3 School of Pharmacy, University of Eastern Finland, Yliopistonranta 1, P.O. Box 1627, FI-70211 Kuopio, Finland; 4 Department of Chemistry, Faculty of Science, University of Maragheh, Maragheh P.O. Box 55181-83111, Iran; ghodratmahmoudi@maragheh.ac.ir (G.M.); ardavan.masoudiasl@gmail.com (A.M.); 5 Independent Radiopharmacy Unit, Faculty of Pharmacy, Medical University of Lublin, 4A Chodźki St., PL-20093 Lublin, Poland; monika.pitucha@umlub.pl

**Keywords:** indole derivatives, kainate receptor ligands, quantum chemical calculations, X-ray studies

## Abstract

The blockade of kainate receptors, in particular with non-competitive antagonists, has—due to their anticonvulsant and neuroprotective properties—therapeutic potential in many central nervous system (CNS) diseases. Deciphering the structural properties of kainate receptor ligands is crucial to designing medicinal compounds that better fit the receptor binding pockets. In light of that fact, here, we report experimental and computational structural studies of four indole derivatives that are non-competitive antagonists of GluK1/GluK2 receptors. We used X-ray studies and Hirshfeld surface analysis to determine the structure of the compounds in the solid state and quantum chemical calculations to compute HOMO and LUMO orbitals and the electrostatic potential. Moreover, non-covalent interaction maps were also calculated. It is worth emphasizing that compounds **3** and **4** are achiral molecules crystallising in non-centrosymmetric space groups, which is a relatively rare phenomenon.

## 1. Introduction

Ionotropic glutamate receptors (iGluRs) are key proteins for synaptic signalling in the central nervous system (CNS), making them promising drug targets [1]. Kainate receptors, belonging to iGluRs, are potential drug targets in schizophrenia, epilepsy, and neurodegenerative diseases [2]. Antagonists of kainate receptors display anticonvulsant and neuroprotective properties. In particular, non-competitive antagonists of kainate receptors, such as ligands of α-amino-3-hydroxy-5-methyl-4-isoxazolepropionic acid (AMPA) receptors, seem attractive due to their better safety profile [3].

As a part of our research on CNS active ligands, we synthesized and studied a series of non-competitive antagonists of kainate GluK1/GluK2 receptors, which are 1,2,3,5-tetrasubstituted indole derivatives with IC_50_ in the low micromolar range [4,5,6]. These compounds belong to the most active GluK1 non-competitive antagonists and are the first reported non-competitive antagonists of the GluK2 receptor. We constructed homology models of investigated receptors to study ligand–receptor interactions at the molecular level [5,7,8]. We also performed experimental and computational structural studies for the most active derivative and its sterically crowded inactive 1-benzylsubstituted analogue [5]. Moreover, we carried out thermal analysis and experimental and computational spectroscopic studies for selected compounds [9].

Here, we report the structures of four compounds with an indole core, supplemented by quantum chemical calculations and Hirshfeld surface analysis. It must be noted that compounds **3** and **4** are achiral molecules that crystallized in non-centrosymmetric space groups. Chirality is an important topic in chemistry, biochemistry, pharmacy and materials science, affecting, in particular, the biological activity of medicinal compounds. However, chiral crystallization of achiral molecules reported here is remarkably uncommon [10,11,12]. It should be stressed that these substances act at the pharmacological receptor as a single molecular entity, an achiral molecule. In fact, by dissolution, the chirality caused by the crystal structure disappears.

## 2. Results and Discussion

### 2.1. Chemistry

**Scheme 1:**
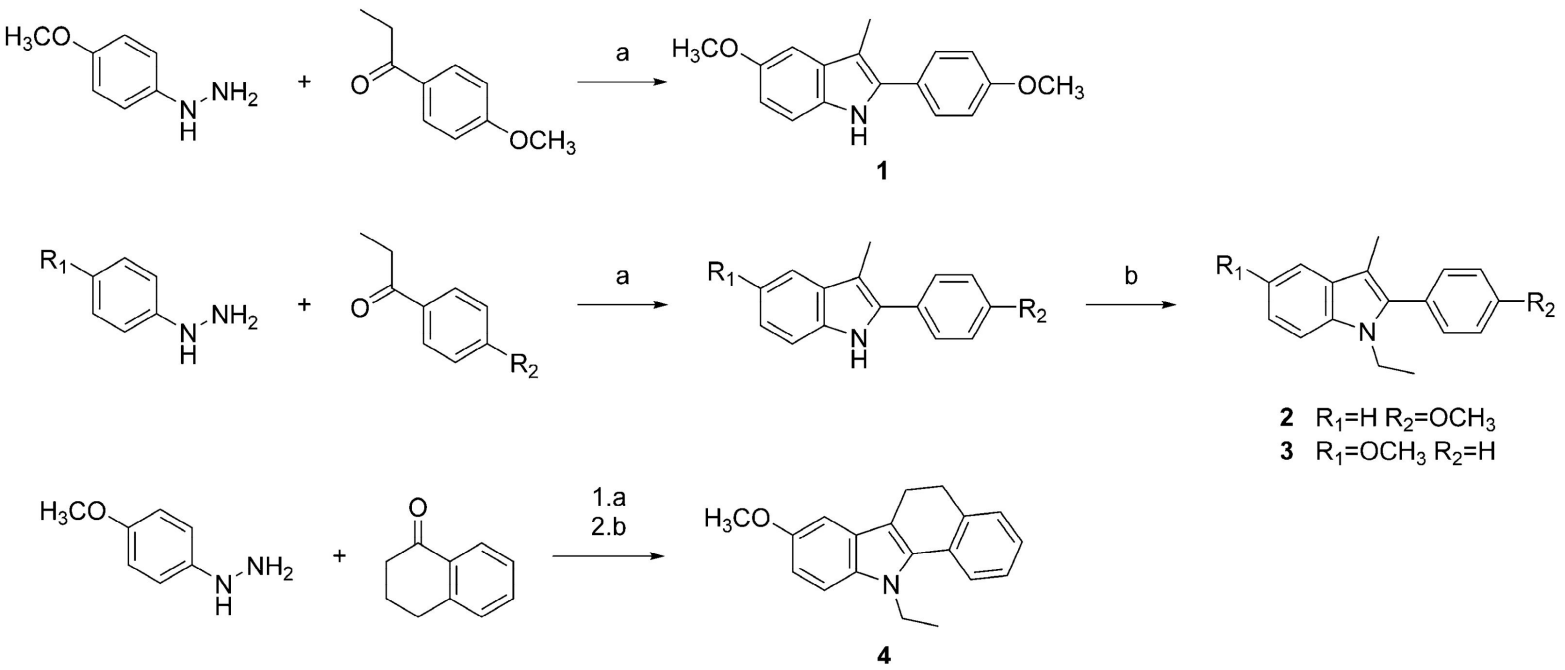
Synthesis of indole derivatives **1**–**4**. Reagents and conditions: (a) EtOH, HCl, reflux; (b) EtI, NaH, DMF.

Four indole derivatives were synthesized employing Fisher’s method, as previously described (Scheme 1) [4]. Shortly, an appropriate phenylhydrazine was refluxed with a ketone under acidic conditions. N-alkylation was affected with ethyl iodide, and the final compounds were purified by recrystalliation from ethanol.

### 2.2. X-ray Studies

**Figure 1:**
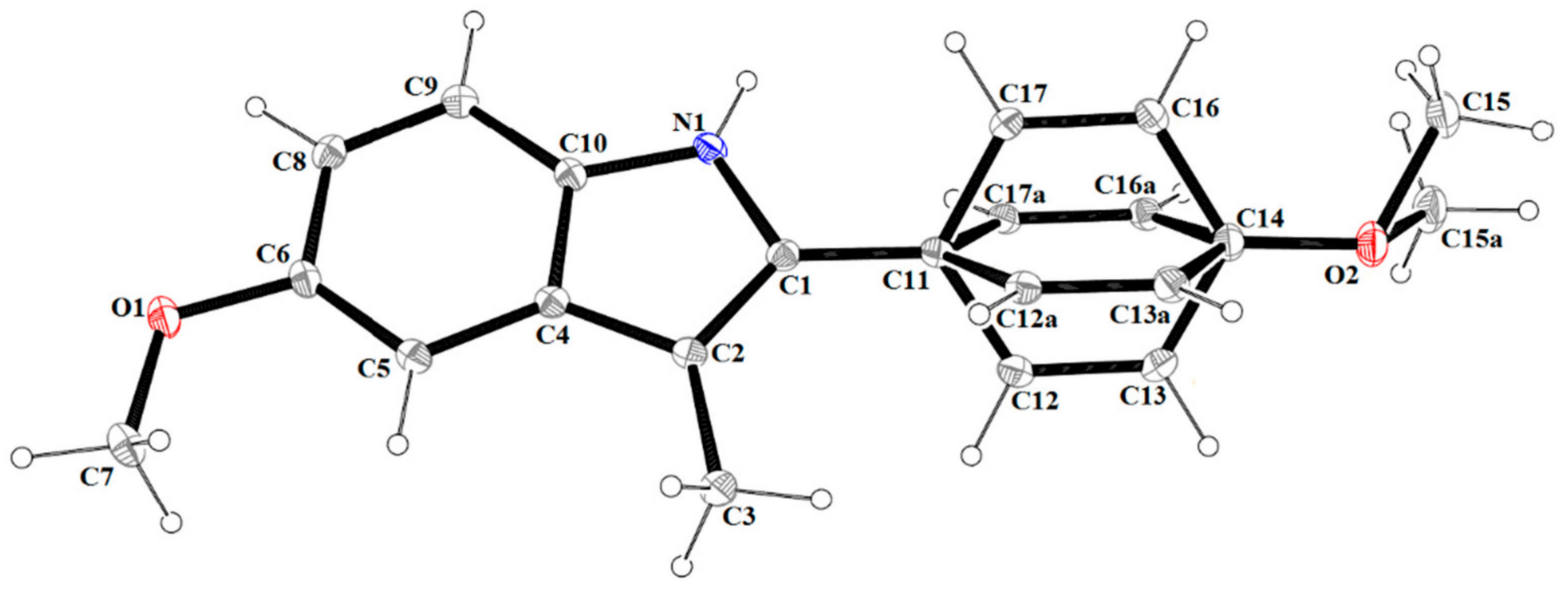
The molecular structure of **1**, with atom labelling. Displacement ellipsoids are drawn at the 30% probability level.

**Figure 2:**
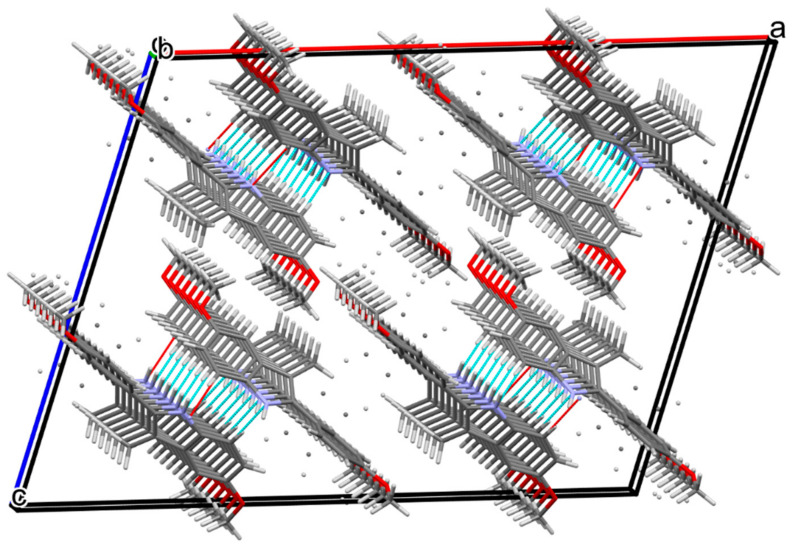
Crystal packing for compound **1** showing hydrogen bonds as dashed blue lines.

**Figure 3:**
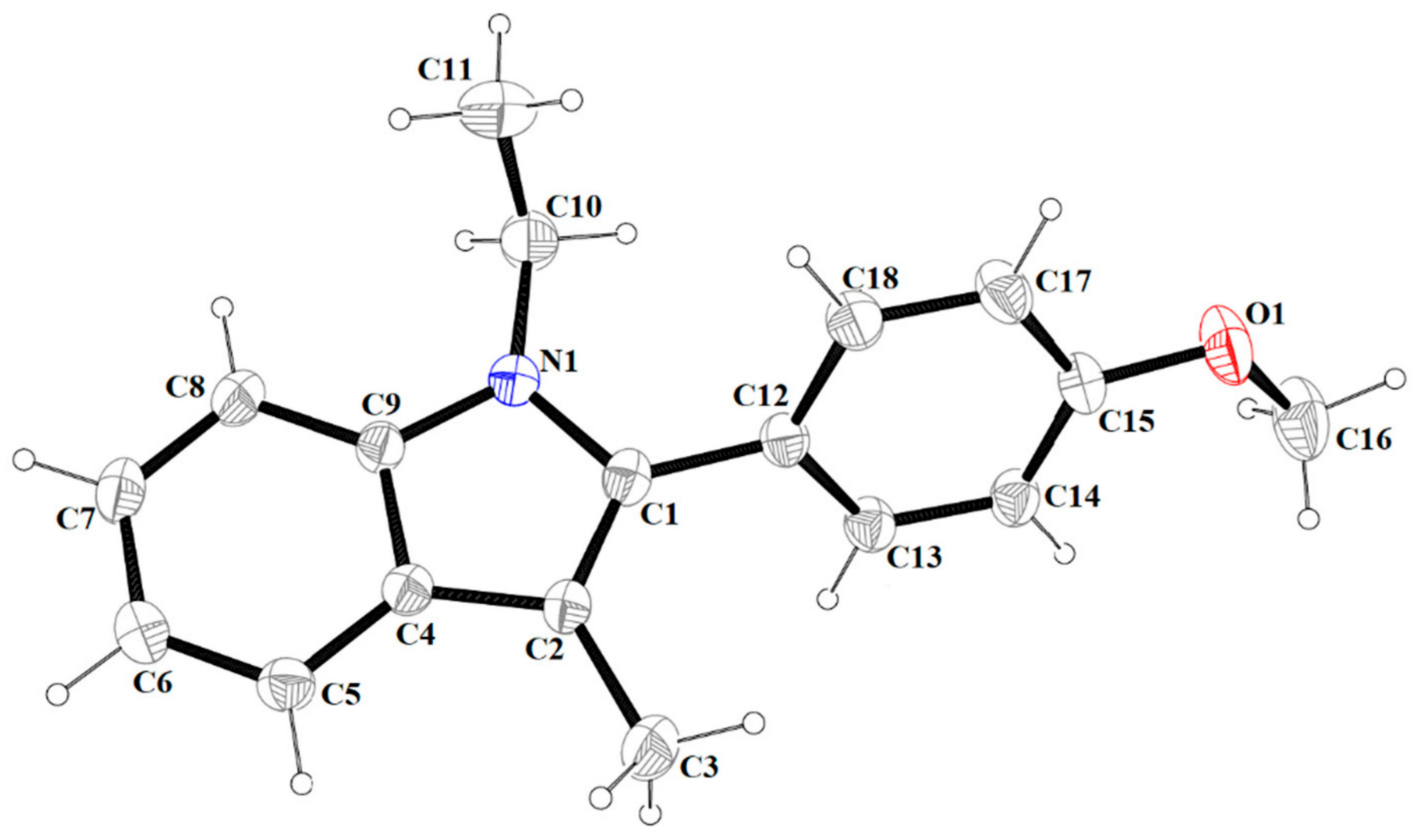
A view of asymmetric unit of **2**, showing the atom-numbering scheme. Displacement ellipsoids are drawn at the 30% probability level.

**Figure 4:**
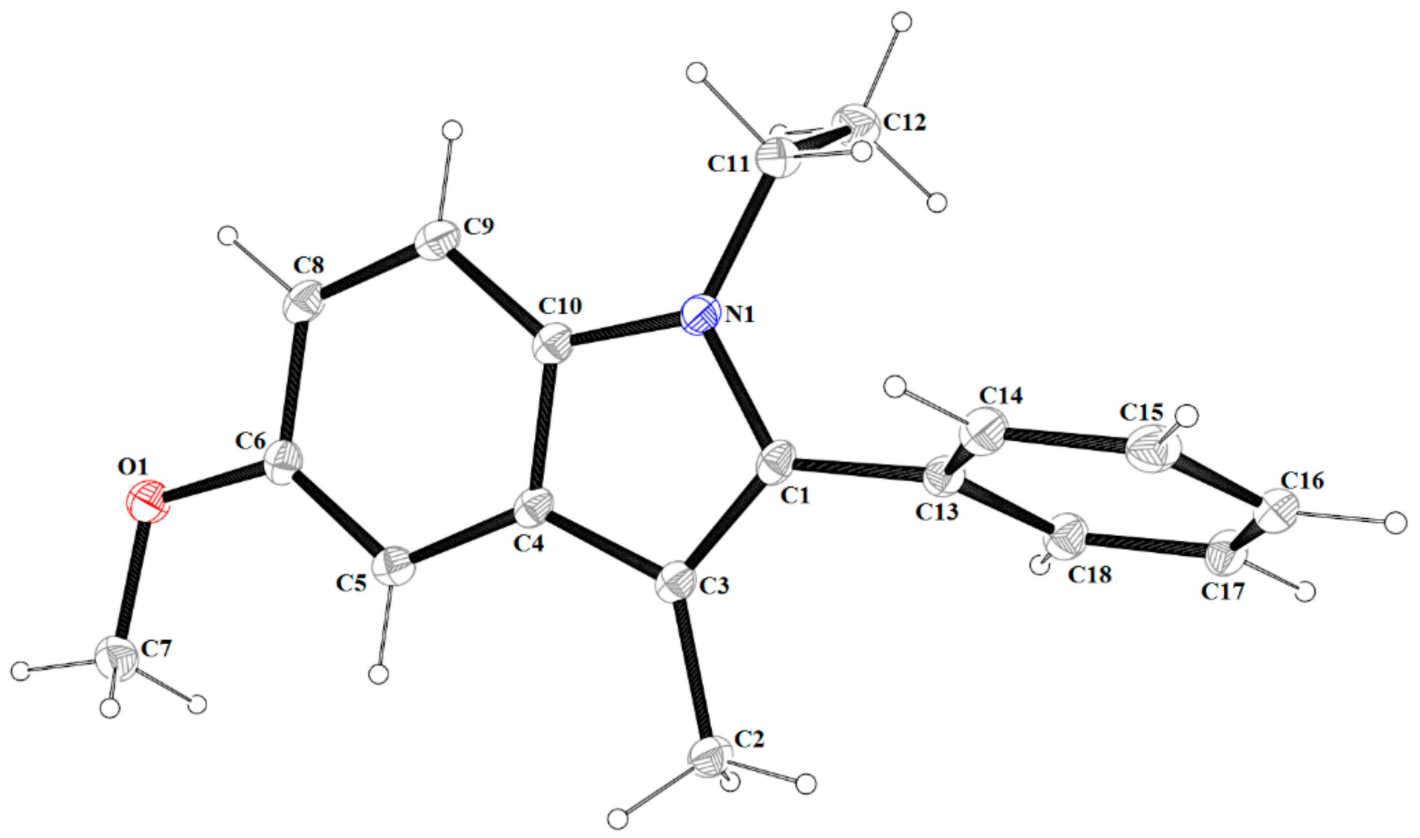
A view of asymmetric unit of **3**, showing the atom-numbering scheme. Displacement ellipsoids are drawn at the 30% probability level.

**Figure 5:**
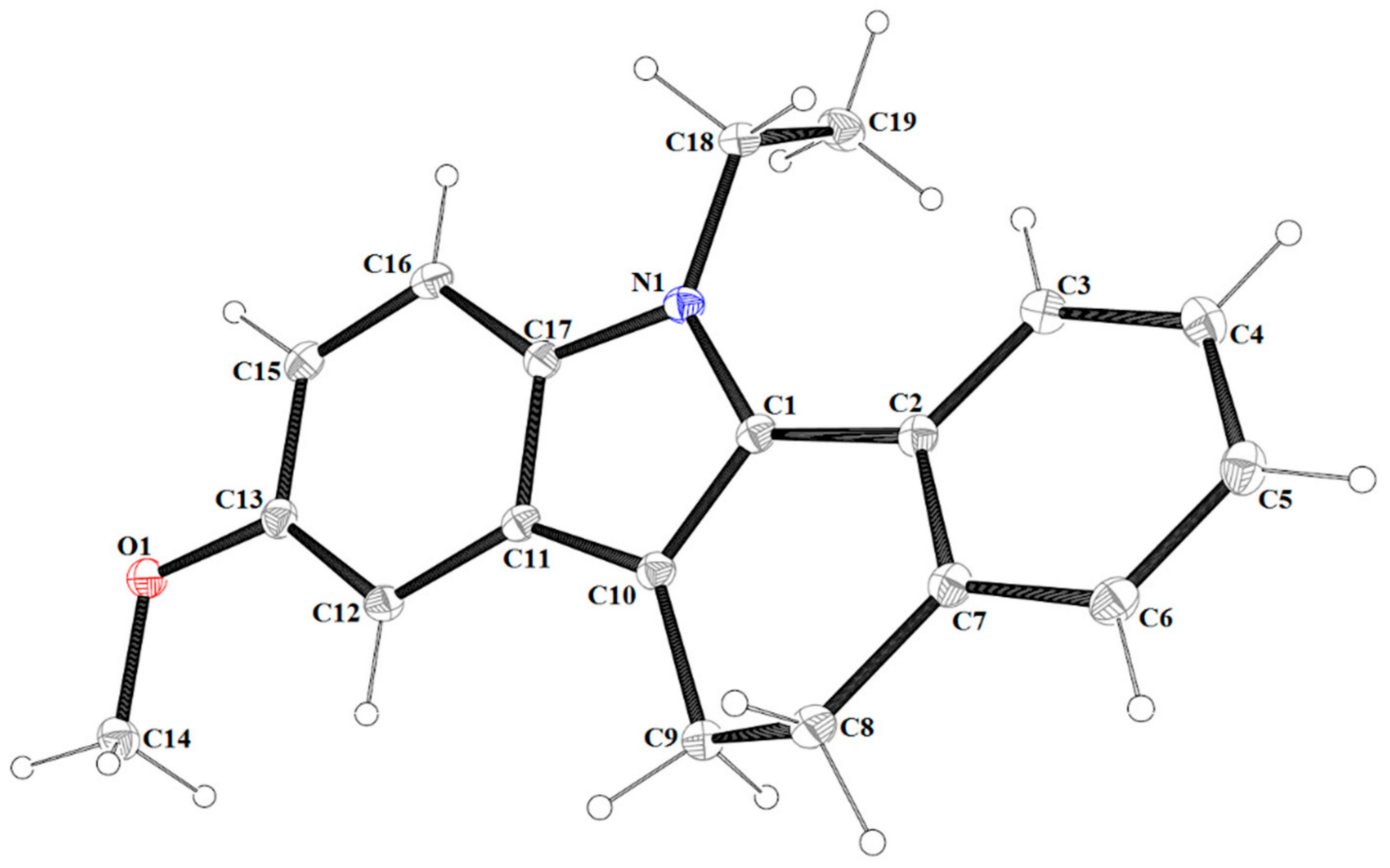
The molecular structure of **4** with atom labelling. Displacement ellipsoids are drawn at the 30% probability level.

**Figure 6:**
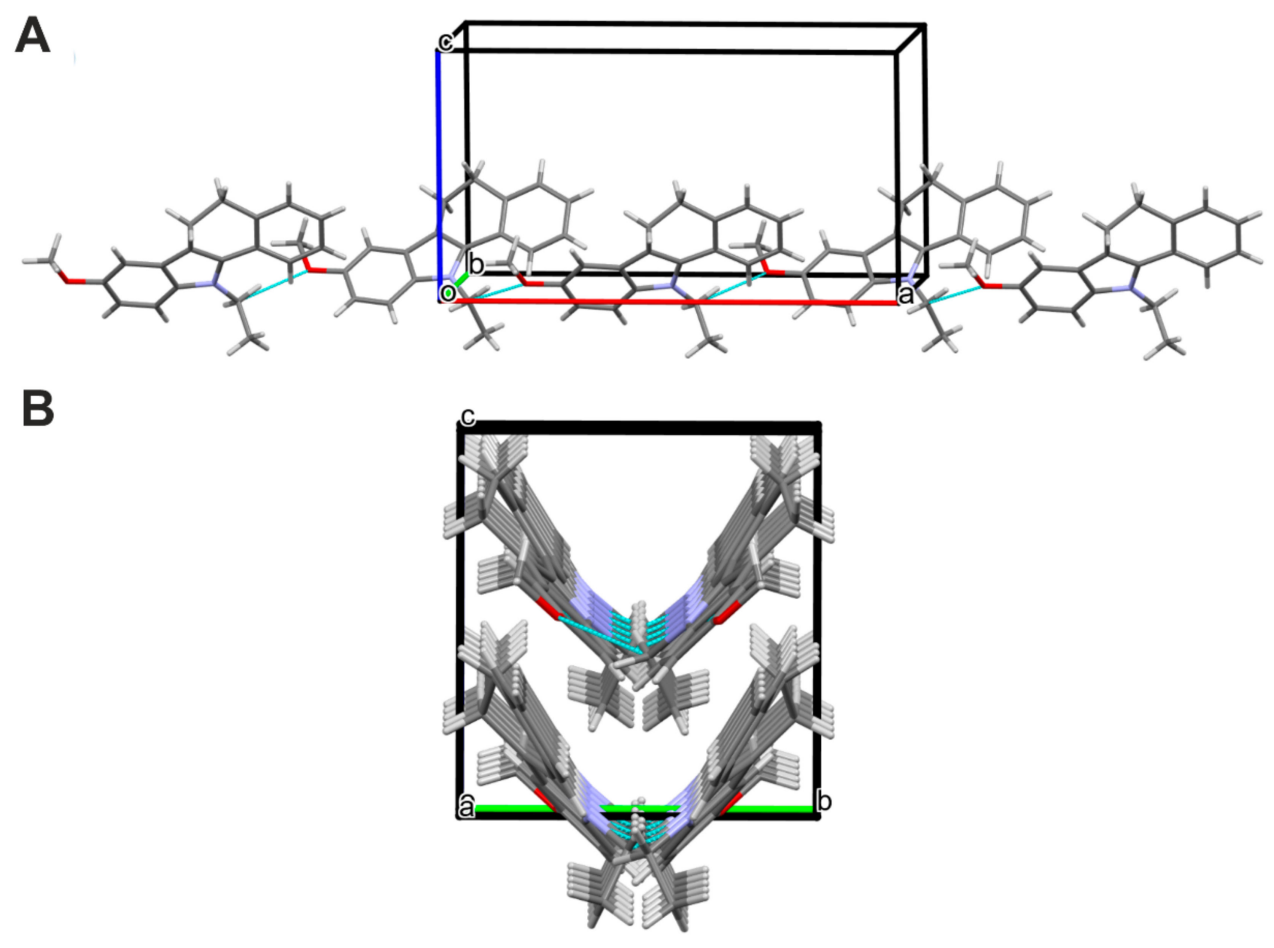
(**A**)—Part of the crystal structure of **4**, showing a molecular chain, which propagates parallel to the *a*-axis. (**B**)—Part of the crystal structure of **4**, showing the V-shaped chains’ architecture.

The results of the X-ray analysis of compounds **1**–**4** are presented in Figure 1, Figure 2, Figure 3, Figure 4, Figure 5 and Figure 6, and the selected bond distances and bond angles are given in Table S1 (Supplementary Materials). Bond distances and angles are in the expected ranges [13] and are comparable with those observed for the other closely related indole derivatives [5,14,15,16,17]. The hydrogen bond parameters are listed in Table 1.

Compound **1** crystallizes in the monoclinic space group *C*2/*c*. The asymmetric unit consists of one molecule of the compound (Figure 1). The indole unit system is planar with an r.m.s deviation of 0.005 Å and a maximum deviation of −0.011(1) Å for atom C2. The 4-methoxyphenyl substituent is disordered over two sites, with occupancy factors in a 0.498(2):0.502(2) ratio. The mean plane through the fused ring system makes dihedral angles of 35.00(2)° and 44.61(5)° with the phenyl rings (C11/C12/C13/C14/C16/C17) and (C11/C12A/C13A/C14/C16A/C17A). The crystal packing along the *c*-axis of compound **1** is illustrated in Figure 2. The molecules of compound **1** are linked by C(9)-H(9)···N(1) hydrogen bonds into one-dimensional columns lying parallel to the [001] direction.

The molecular structure of the compound **2** is illustrated in Figure 3. Similar to **1,** compound **2** crystallizes in the monoclinic system but in space group *P*2_1_/*n*. The asymmetric unit contains a single molecule. The molecule features methyl, ethyl and 4-methoxyphenyl groups attached to an indole moiety. The indole unit is planar with an r.m.s deviation of 0.011 Å and a maximum deviation of −0.018(5) Å for atom C2. The whole molecule of compound **2** is non-planar. The C11 atom of the ethyl group lies below the indole unit plane (−1.208(4) Å). Additionally, the dihedral angle between the planes formed by non-hydrogen atoms of the indole fragment and the phenyl ring (C12–C18) is 60.93(7)°.

Compound **3** crystallizes in non-centrosymmetric space group *P*2_1_2_1_2_1_, and the asymmetric unit contains a single molecule (Figure 4). The indole fragment is an essential planar with an r.m.s deviation of 0.021 Å and a maximum deviation of −0.035(1) Å for the N1 atom. Similar to the previously mentioned compounds **1** and **2**, the molecule of **3** is also non-planar. In the molecule, the phenyl (C13–C18) ring forms dihedral angles of 66.71(5)° with the mean plane of the central indole moiety. The ethyl group lies above the plane of fused rings (C(11): 0.205(3) Å and C(12): 1.682(3) Å).

The atom-numbering scheme and molecular structure of **4** are shown in Figure 5. The compound **4** crystallizes in non-centrosymmetric space group *Pbc*2_1_, and the asymmetric unit contains a single molecule. For compound **4**, the indole core is planar with a maximum deviation of −0.030(2) Å for C11. The molecules of **4** are linked via C(18)-H(18A)∙∙∙O(1) hydrogen bonds into infinite V-shape chains (Figure 6) running parallel to the [100] direction.

### 2.3. Hirshfeld Surface Analysis

The Hirshfeld surface of a molecule in a crystal is computed by partitioning space in the crystal into regions where the electron distribution of a sum of spherical atoms for the molecule dominates the corresponding sum over the crystal [18]. The Hirshfeld surface defines the space occupied by a molecule in a crystal for the purpose of partitioning the crystal electron density into molecular fragments [19].

**Figure 7:**
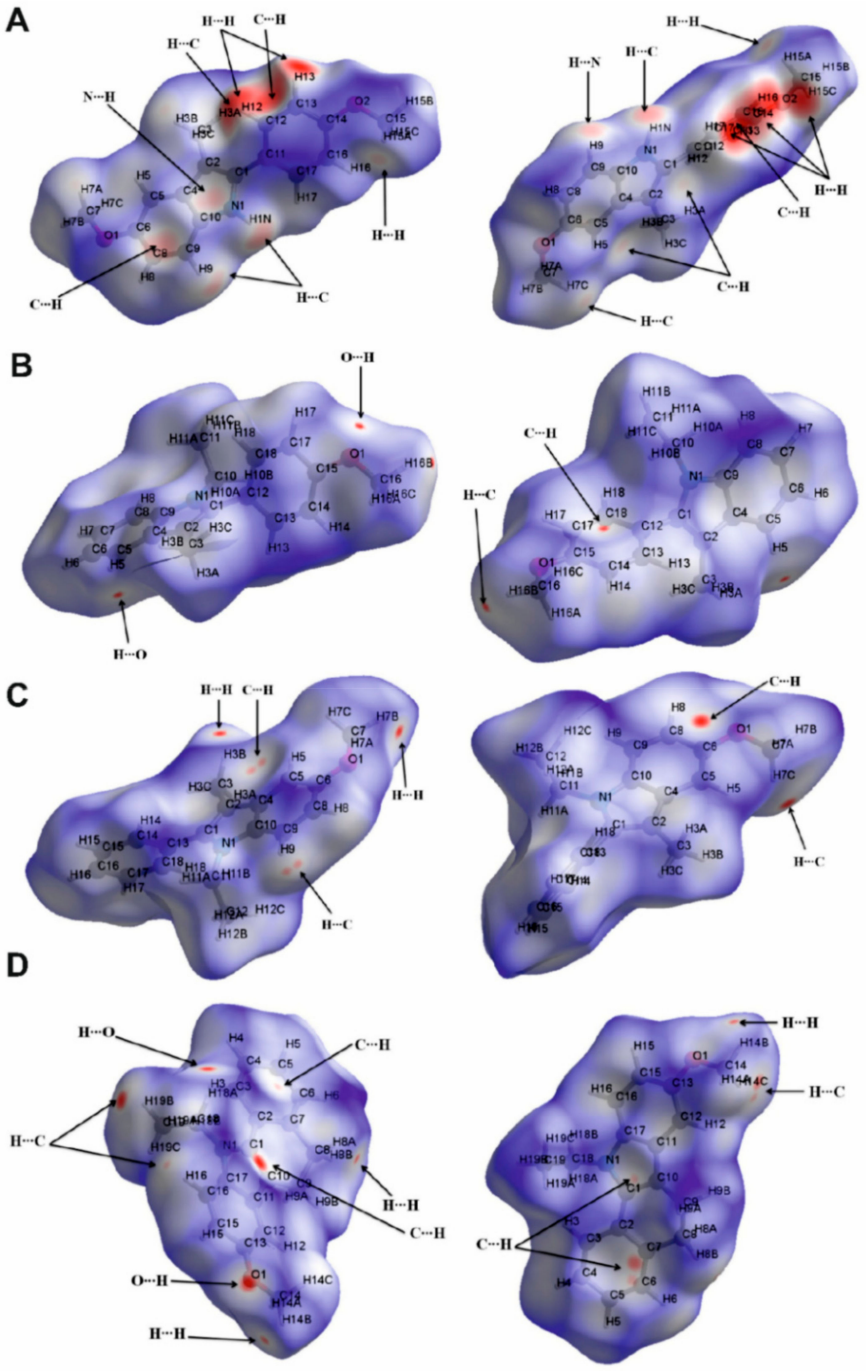
Two views of Hirshfeld surfaces mapped over *d_norm_* for compounds **1** (**A**), **2** (**B**), **3** (**C**) and **4** (**D**).

**Figure 8:**
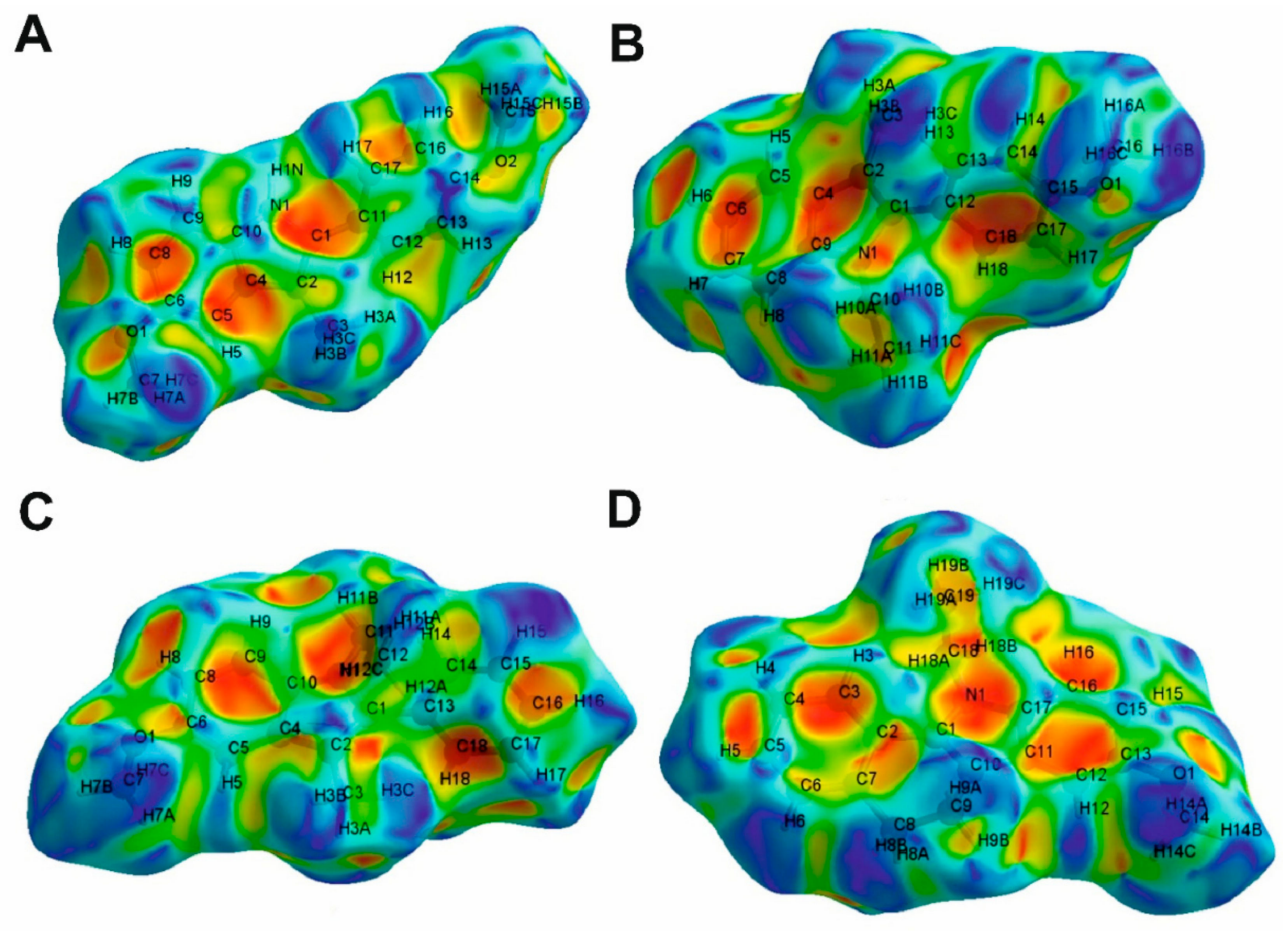
Hirshfeld surface mapped over *shape index* function for compounds **1** (**A**), **2** (**B**), **3** (**C**) and **4** (**D**).

The Hirshfeld surfaces mapped over *d_norm_* and the *shape index* are represented in Figure 7 and Figure 8, respectively. The surfaces are represented as transparent to allow the visualization of the molecular moiety around which they were calculated.

On the *d_norm_* surface of compound **1** (Figure 7A), there are several prominently interconnected red areas around the phenyl ring, including C11-C16 atoms that are attributed to the strong H···H and H···C interactions observed in crystal packing. H···C/C···H interactions are also observable as light red areas around H1N, C8 and C9 atoms. On the *shape index* surface of compound **1** (Figure 8A), H···C/C···H close contacts, attributed to C–H···π interactions, appear as the convex blue areas around hydrogen atoms (H···C interactions) and the concave orange areas above aromatic rings (C···H interactions). Additionally, two pale red spots around H9 and N1 are imputed to nonclassical C–H···N hydrogen bonds.

The *d_norm_* surface of compound **2** (Figure 7B) consists of mostly white areas with four red spots. Two of these points are due to the formation of nonclassical C–H···O hydrogen bond between O1 and H5 atoms. Two more points observed around H16B and methoxy phenyl are attributed to the formation of C–H···π interactions. These interactions are seen as the convex blue and the concave orange areas (Figure 8B).

Hirshfeld surface mapped over *d_norm_* for compound **3** (Figure 7C) shows that the intermolecular H···C/C···H interactions have the dominant role in the structure of this compound. The red spots on two sides of the aromatic ring, including C4, C5, C6, C8, C9 and C10 atoms, are due to the participation of this ring in the formation of two C–H···π interactions with H7C and H9 atoms. Additionally, H···H close contacts appear as two red spots around H3B and H7B.

The *d_norm_* surface of compound **4** (Figure 7D) contains several red spots. The majority of these points are related to the intermolecular H···C/C···H interactions. Additionally, two distinct red spots around O1 and H18A are due to nonclassical C–H···O hydrogen bonds.

**Figure 9:**
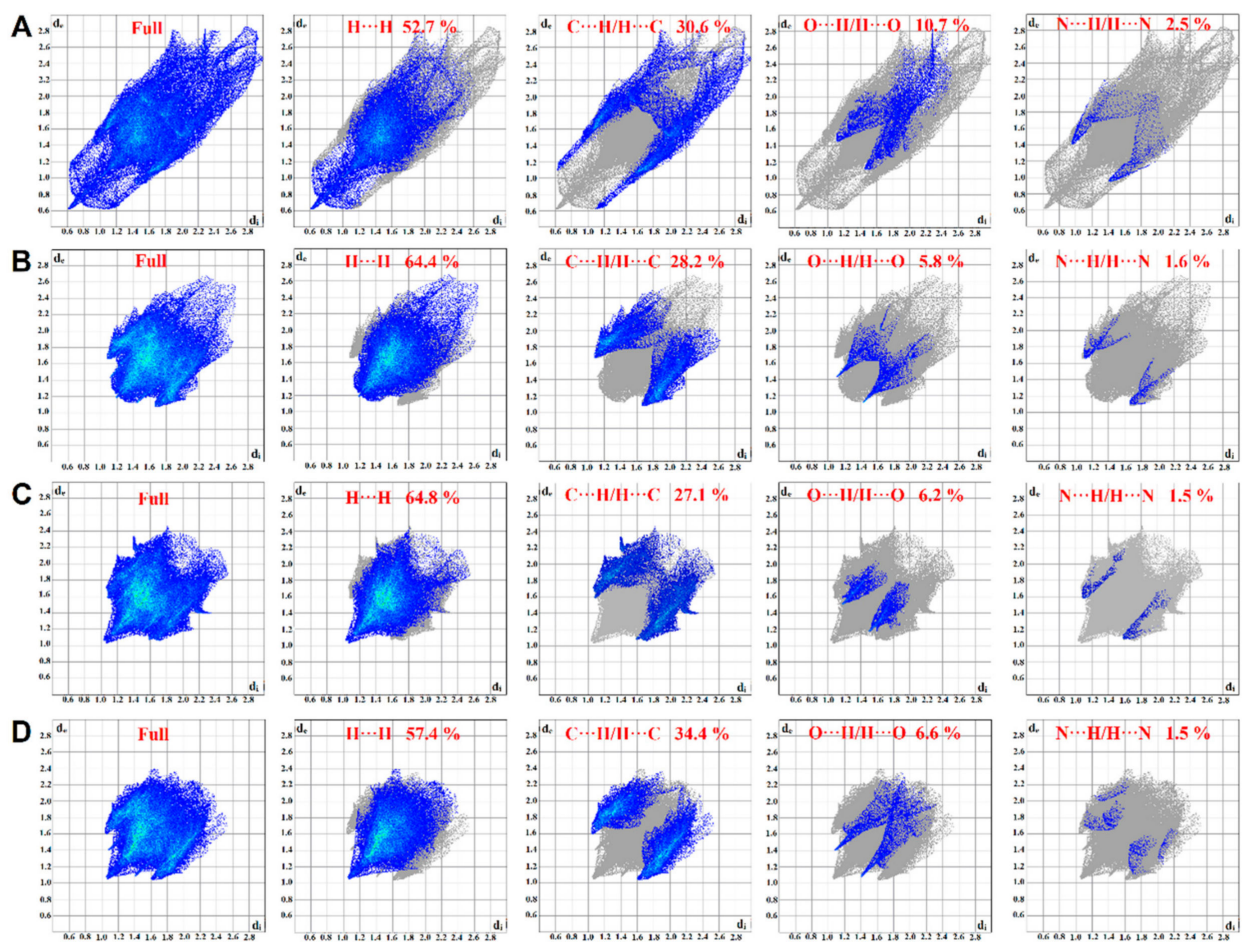
The 2D fingerprint plots for compounds **1** (**A**), **2** (**B**), **3** (**C**) and **4** (**D**).

To quantify the intermolecular interactions, 2D fingerprint plots of Hirshfeld surfaces were used. In these plots (Figure 9), complementary regions are visible in the upper and bottom parts of plots where one molecule acts as a donor (*d_e_* > *d_i_*) and the other as an acceptor (*d_e_ < d_i_*). The quantitative analysis of interactions reveals that the highest share of the total Hirshfeld surface for all four compounds belongs to the H···H close contacts with the share of 52.7%, 64.4%, 64.8% and 57.4% for compounds **1**, **2**, **3** and **4**, respectively. This high share can be attributed to the high number of hydrogen atoms and their spatial position in the structure of compounds [20]. The intermolecular H···C/C···H interactions have the second share of the total surface for all four compounds with the share of 30.6%, 28.2%, 27.1% and 34.4% for compounds **1**, **2**, **3** and **4**, respectively, and appear as wings on the bottom right (C···H interaction) and top left (H···C interaction) of the 2D fingerprint plot. These two interactions comprise 83.3%, 92.6%, 91.9% and 91.8% for compounds **1**, **2**, **3** and **4**, respectively. The remaining portion of the total surface mostly belongs to H···O/O···H and H···N/N···H interactions with 10.7% and 2.5% for compound **1**, 5.8% and 1.6% for compound **2**, 6.2% and 1.5% for compound **3** and 6.6% and 1.5% for compound **4**, respectively.

### 2.4. Quantum Chemical Calculations and NCI Analysis

Quantum chemical calculations were used to calculate HOMO and LUMO orbitals and electrostatic potential surface. X-ray structures were used as the starting conformation for calculations. RMSD between the X-ray and computed structures: 0.1555, 0.2170, 0.1944 and 0.2324 Å for compounds **1**, **2**, **3** and **4**, respectively.

**Figure 10:**
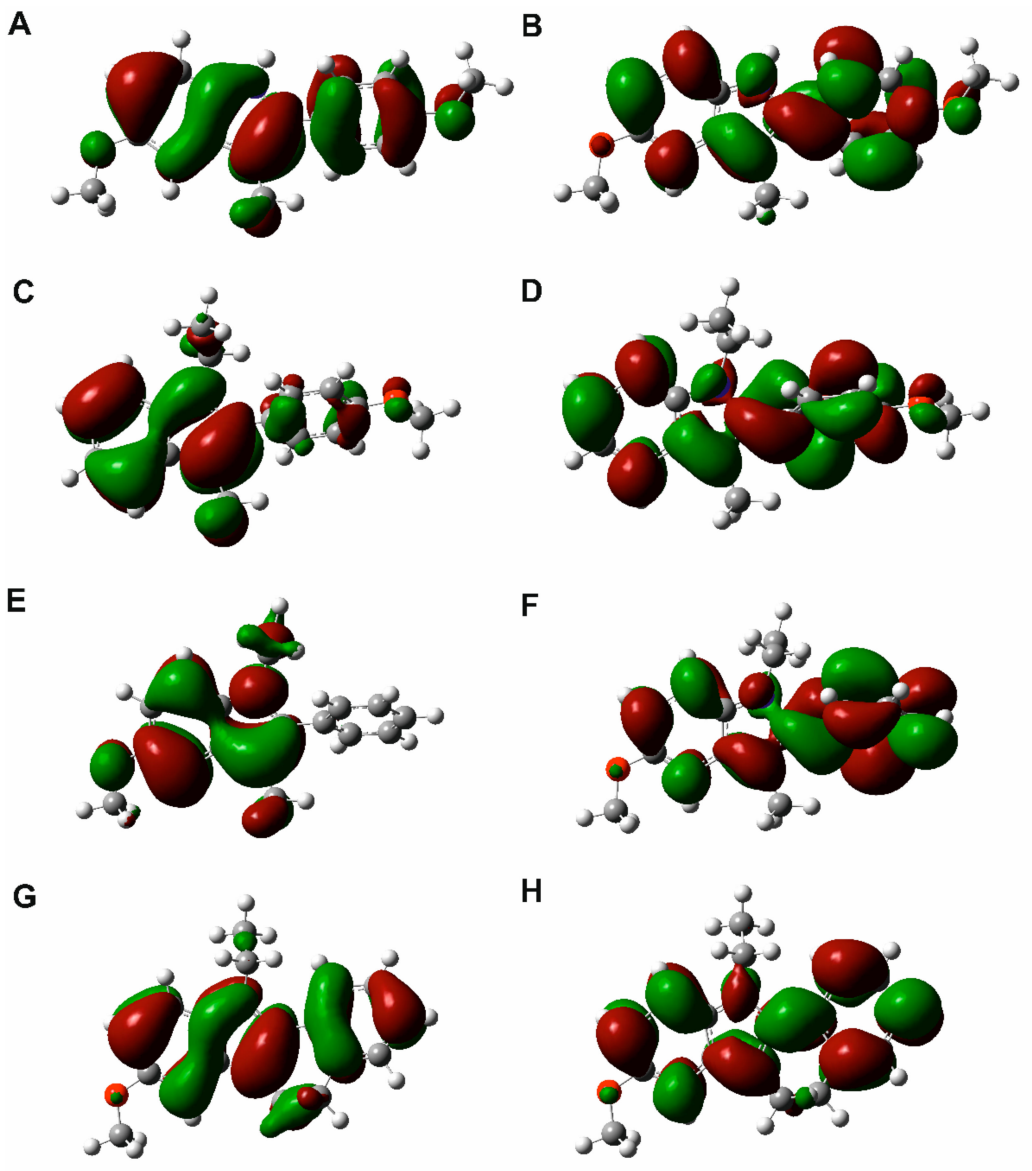
The shapes of HOMO (**A**,**C**,**E**,**G**) and LUMO (**B**,**D**,**F**,**H**) orbitals for compounds **1** (**A**,**B**), **2** (**C**,**D**), **3** (**E**,**F**) and **4** (**G**,**H**).

HOMO and LUMO orbitals surfaces are shown in Figure 10, and HOMO and LUMO orbitals energies accompanied by the HOMO-LUMO gap are collected in Table 2.

Frontier Molecular Orbital (FMO) theory is an important model to describe the reactivity of chemical compounds, which focuses on HOMO and LUMO orbitals. FMO analysis is broadly used to explain the optical and electronic properties of organic compounds [21]. As was already stated, knowledge of the HOMO and LUMO and their energy allows judging the chemical reactivity of molecules [21]. In order to describe molecular interactions, it can be assumed that the LUMO accepts electrons and the HOMO donates electrons. LUMO energy reflects the electron affinity and HOMO energy concerns the ionization potential. The HOMO-LUMO energy gap refers to charge transfer interaction within the molecule and can be applied to determine molecular electrical transport properties [21]. A chemical compound with a high HOMO-LUMO energy gap is characterized by low chemical reactivity and high kinetic stability as it is energetically unfavourable to add an electron to LUMO in order to remove electrons from HOMO [21].

The HOMO surfaces for the studied compounds are mainly located on the indole core for all the compounds and on the additional benzene ring for compounds **1** and **4**. The LUMO surfaces are situated on the indole system and on the phenyl substituent for all the molecules. The location of HOMO and LUMO orbitals may indicate the most reactive parts of the compounds. The values of HOMO and LUMO energies are comparable for all the studied compounds, indicating that the substituents do not have a major effect on this property (see Table 2). Considering the HOMO-LUMO gap, the studied compounds can be considered relatively stable, while the lowest value of this parameter for compound **4** indicates its enhanced chemical reactivity.

**Figure 11:**
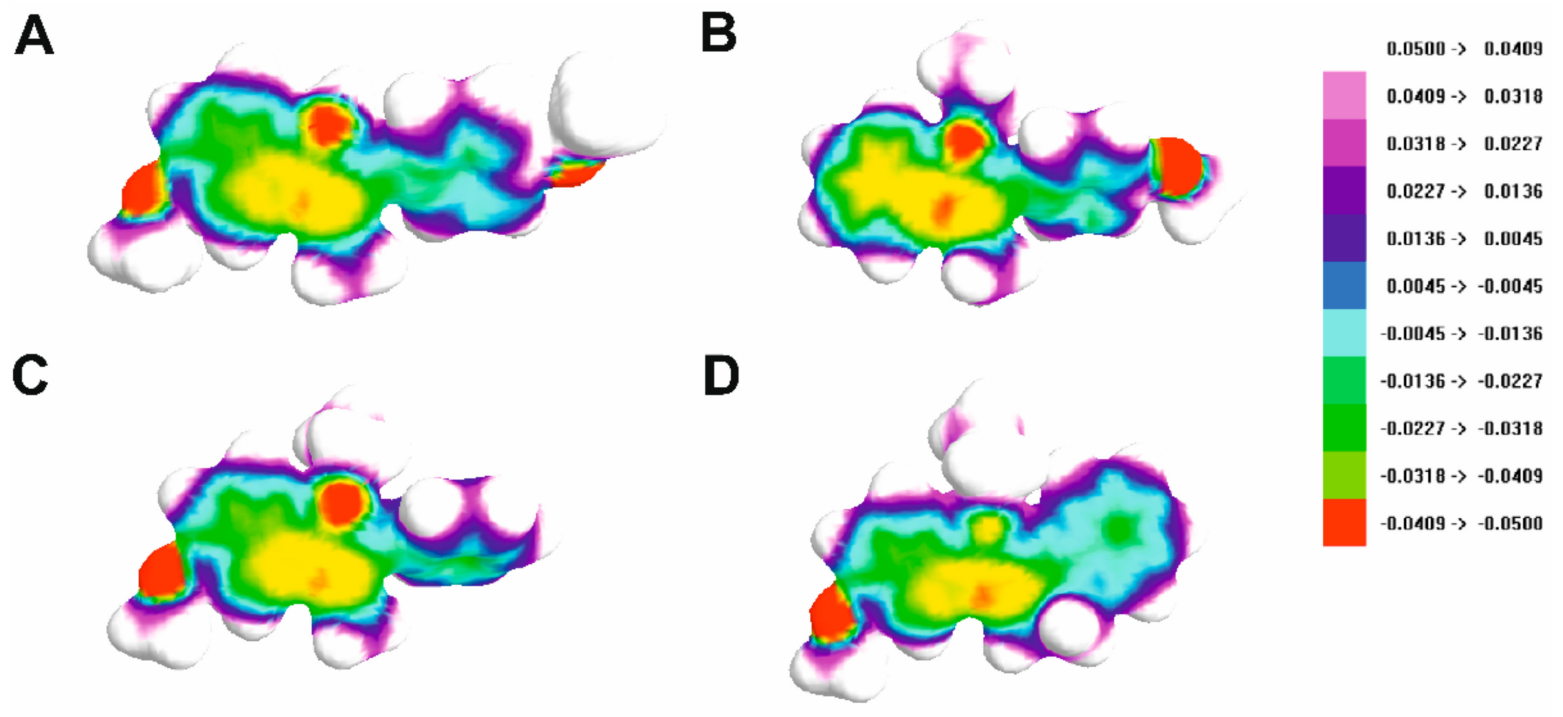
Electrostatic potential surface for compounds **1** (**A**), **2** (**B**), **3** (**C**) and **4** (**D**).

In order to gain deeper insight into the electronic properties of the investigated compounds, electrostatic potential surfaces were calculated and are shown in Figure 11.

Investigation of small molecules’ electrostatic potential surfaces (ESP) is a crucial task in computer-assisted drug design as it is necessary to optimize ligand–protein electrostatic complementarity [22]. It is generally accepted that molecular electrostatics are responsible for a compound’s chemical reactivity and its capability to be involved in molecular interactions [22]. As can be seen in Figure 11, the oxygen and nitrogen atoms of the studied molecules are their most electronegative part, which can interact with the target proteins, as reported previously [5]. Further optimization of the compounds will aim to introduce more anchor points for polar interactions into the novel derivatives.

**Figure 12:**
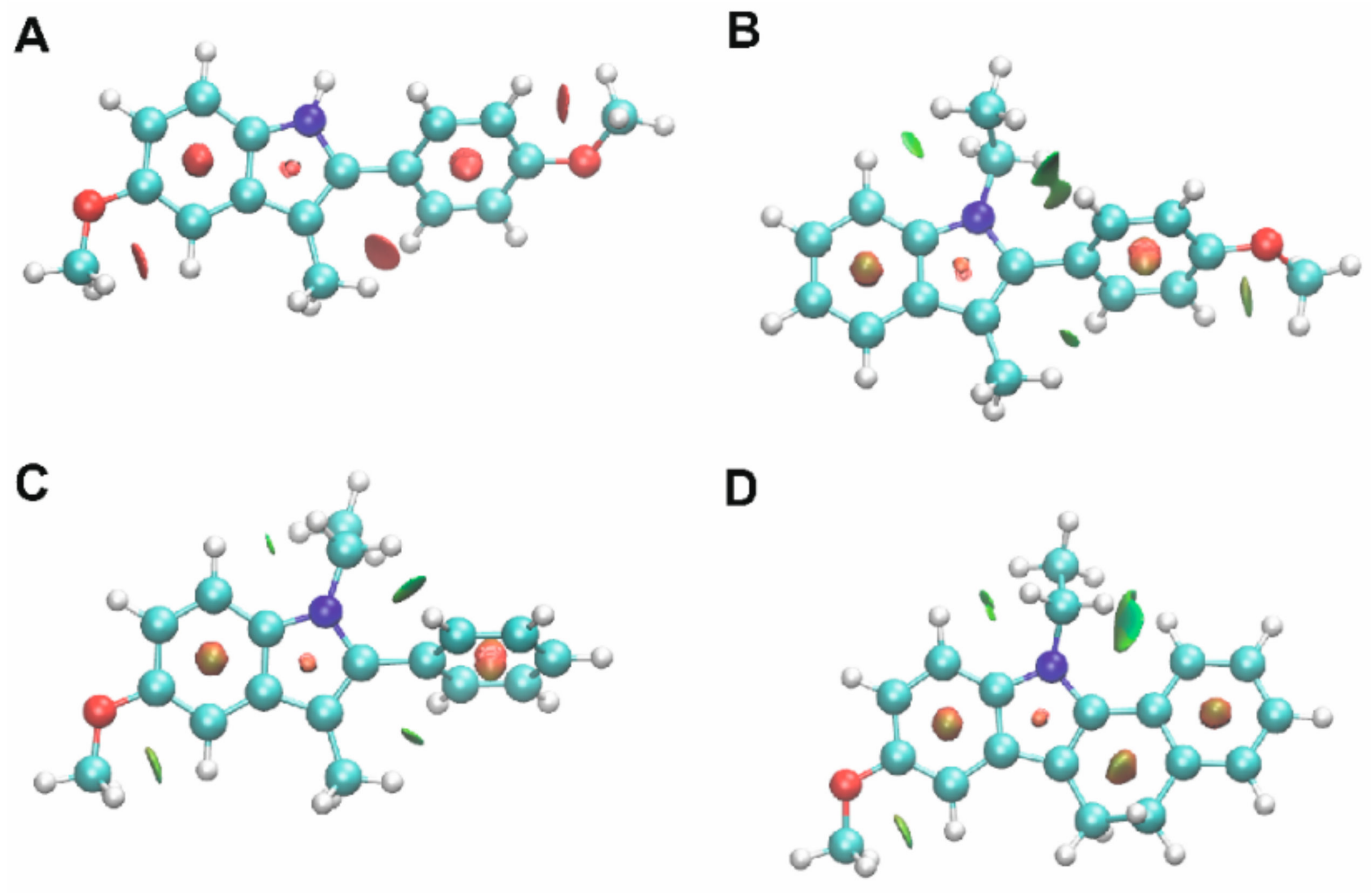
Non-covalent interactions for compounds **1** (**A**), **2** (**B**), **3** (**C**) and **4** (**D**). Green colour denotes weak attractive interactions, while red indicates strong and repulsive interactions.

Finally, non-covalent interactions (NCI) maps were also generated for the studied compounds and are shown in Figure 12.

Non-covalent interactions are crucial for understanding a number of chemical, biological and technological problems [23]. Describing them accurately is important for the process of decoupling the complex balance of forces that are responsible for molecular interactions [23]. In particular, molecular stability is maintained by weak inter- and intramolecular interactions [24]. Deciphering non-covalent interactions is crucial in drug design as they are responsible for ligand–protein interactions. It can be seen in Figure 12 that a number of weak attractive interactions (shown in green) are maintained by the investigated compounds, in particular between alkyl groups and aromatic hydrogen atoms. The observed differences may be caused, among others, by the structure of the molecule as well as the type, amount and position of the appropriate substituents. For example, one of the substituents of the indole unit in molecule 4 makes the compound roughly planar (excluding the ethyl group). In compound **3**, the phenyl ring is twisted in relation to the indole plane and can be a steric hindrance. Both compounds have a methoxy group connected to the benzene ring of the indole system, but weak C-H ··O hydrogen interactions are only observed in the case of compound 4. The pattern of non-covalent interactions can be applied to design the next series of derivatives of the studied compounds.

## 3. Materials and Methods

### 3.1. Chemistry

Compounds **1**–**4** were synthesized following the previously reported methodology [4]. Detailed spectral characterization of these compounds can be found elsewhere [4,9].

### 3.2. X-ray Studies

An Oxford Diffraction Xcalibur CCD diffractometer with graphite-monochromated MoKα radiation (λ = 0.71073 Å) was used for diffraction data collection. The measurements were carried out at a temperature of 298 K or 100 K. The CrysAlis [25] suite of programs was used for data collection, cell refinement and data reduction. A multiscan absorption correction was applied. The structures were solved by direct methods using SHELXS-2018 and refined by the full-matrix least-squares on F2 using the SHELXL-2018 [26] (both operating under WinGX) [27]. All non-hydrogen atoms were refined with anisotropic displacement parameters. The H-atoms attached to carbon were positioned geometrically and refined applying the riding model [C−H = 0.93–0.98 Å and with Uiso(H) = 1.2 or 1.5 Ueq(C)]. The H atom on N atom (compound **1**) was located in a different Fourier map and was refined isotropically. The chemicals’ absolute configurations of compounds **3** and **4** could not be determined unambiguously because a heavy atom is not present in structures. For this reason, the absolute structure parameters were meaningless with rather poor accuracy. Parameters for data collection and structure refinement data for all structures are summarized in Table 3. The molecular plots were drawn with ORTEP3 for Windows [27] and Mercury [28]. The geometrical calculations were performed using PLATON program [29]. The CIF file refinement can be retrieved from the Cambridge Crystallographic Data Center (CCDC).

### 3.3. Hirshfeld Surface and Fingerprint Analysis

The CrystalExplorer [18] computer program was used for the preparation of molecular Hirshfeld. These surfaces are mapped using the normalized contact distance (*d_norm_*), which is calculated using the following equation:

$$d_{norm}=\frac{d_{i}-r_{i}^{vdw}}{r_{i}^{vdw}}+\frac{d_{e}-r_{e}^{vdw}}{r_{e}^{vdw}}$$

where *d_e_* is the distance from the Hirshfeld surface to the nearest atom outside the surface, *d_i_* is the distance from the Hirshfeld surface to the nearest atom inside the surface and *d_norm_* is defined in terms of de and di and the van der Waals (vdW) radii of atoms. Three-dimensional (3D) Hirshfeld surface maps are generated with *d_norm_* using a red–white–blue colour scheme, indicating shorter contacts, vdW contacts, and longer contacts, respectively, and two dimensional (2D) fingerprint plots are generated using *d_e_* and *d_i_*.

### 3.4. Quantum Chemical Calculations and NCI Analysis

B3LYP DFT method and the 6-311++G(2df, 2pd) basis set of Gaussian09 [30] software GaussView v. 6.0 were used to visualize HOMO and LUMO orbital shapes and to compute their energies, as previously reported [17]. Electrostatic potential distribution was calculated and visualized with ArgusLab v. 4.0.1 [31]. Non-covalent interactions maps were computed with NCIPlot v. 3.0 [23] and visualized with VMD v. 1.9.4 [32], as reported earlier [17,33]. X-ray structures of compounds **1**–**4** were used as a starting point in all the calculations. Molecular alignment of X-ray and modelled structures was performed with Discovery Studio Visualizer v. 17.1.0.16143, and RMSD was calculated using Schrödinger suite of software release 2022-1.

## 4. Conclusions

Here, we report experimental and computational structural studies of four indole derivatives which are non-competitive antagonists of kainate GluK1/GluK2 receptors. The indole moiety is an essential planar in all presented structures. The main finding of this study is that compounds **3** and **4** are achiral molecules crystallizing in non-centrosymmetric space groups, which is a rare phenomenon. The origin of the noncentrosymmetry of crystal structures may be found in the electronic asymmetry of **3** and **4** and in the packing of the molecular units. The unit cells of these compounds consist of four molecules that form helical arrangements. Additionally, in the case of **4**, due to weak hydrogen bonds, the V- shape chains architecture across a two-fold rotation axis is also observed. However, it should be stressed that achiral molecules which form chiral crystals act at their biological targets as achiral entities, so the reported phenomenon has no biological relevance but may be important in materials science.

Hirshfeld surface analysis indicates the effective role of aromatic rings, indolic nitrogen and methoxy functional group in the directionality and organization of the crystal packing of compounds. The intermolecular H···C/C···H interactions have the second share of the total Hirshfeld surface and appear as distinct red spots over *d_norm_* surfaces for all four compounds. The non-covalent interactions (NCI) maps demonstrate a number of weak attractive interactions by the investigated compounds, in particular between alkyl groups and aromatic hydrogen atoms. The HOMO surfaces for the studied compounds are mainly located on the indole core for all the compounds and on the additional benzene ring for compounds **1** and **4**. The LUMO surfaces are situated on the indole system and on the phenyl substituent for all the molecules. The location of HOMO and LUMO orbitals may indicate the most reactive parts of the compounds. Considering the HOMO-LUMO gap, the studied compounds can be considered relatively stable, while the lowest value of this parameter for compound **4** indicates its enhanced chemical reactivity. Based on the electrostatic potential surfaces, the oxygen and nitrogen atoms of the studied molecules are their most electronegative part, which can interact with the target proteins.

The performed detailed structural characterization of the compounds may be useful for the computer-assisted design of the next series of derivatives of the studied compounds.

## Figures and Tables

**Table 1:** Hydrogen bonding geometry.

D-H···A	d (D-H)	d (H···A)	d (D···A)	∠ DHA
**1**				
C(9)-H(9)∙∙∙N(1) ^1a^	0.93	2.52	3.449(2)	174
**4**				
C(18)-H(18A)∙∙∙O(1) ^4a^	0.99	2.56	3.430(3)	146

Symmetry codes: compound **1**: (1a) x + 1/2, y − 1/2, −z + 1/2; compound **4**: (4a) x + 1/2, −y + 1, z.

**Table 2:** Energies of HOMO and LUMO orbitals for compounds **1**–**4**.

Compound	E_HOMO_ (eV)	E_LUMO_ (eV)	HOMO-LUMO Gap (eV)
**1**	−5.30	−0.91	4.39
**2**	−5.31	−0.75	4.56
**3**	−5.36	−0.99	4.37
**4**	−5.26	−1.21	4.05

**Table 3:** Crystal data and structure refinement of **1**–**4**.

Compound	1	2	3	4
**Temperature K**	298(2)	298(2)	100(2)	100(2)
**Empirical formula**	C_17_H_17_NO_2_	C_18_H_19_NO	C_18_H_19_NO	C_19_H_19_NO
**Formula weight**	267.31	265.34	265.34	277.35
**Crystal system**	monoclinic	monoclinic	orthorhombic	orthorhombic
**Space group**	*C*2/*c*	*P*2_1_/*n*	*P*2_1_2_1_2_1_	*Pbc*2_1_
**a (Å)**	27.464(3)	9.2916(7)	7.6910(3)	17.2868(5)
**b (Å)**	4.9385(4)	8.7458(5)	7.8087(3)	8.7543(3)
**c (Å)**	20.956(2)	18.6641(13)	23.5793(10)	9.4312(2)
**α (°)**		90.00	90	90
**β (°)**	108.564(11)	102.484(7)	90	90
**γ (°)**		90.00	90	90
**Volume (Å^3^)**	2694.4(5)	1480.83(18)	1416.10(10)	1427.26(7)
**Z**	8	4	4	4
**μ (mm^−1^)**	0.086	0.073	0.077	0.079
**Absorption correction**	multi-scan	multi-scan	multi-scan	multi-scan
**F(000)**	1136	568	568	592
**Crystal size (mm)**	0.10 × 0.15 × 0.40	0.13 × 0.27 × 0.50	0.40 × 0.40 × 0.50	0.28 × 0.30 × 0.30
**θ range (°)**	2.95 to 26.02	2.58 to 27.10	2.75 to 26.01	3.20 to 27.09
**Reflections collected/unique**	5674/2615	10931/3261	8686/2778	9669/3008
**R_int_**	0.0289	0.0427	0.0361	0.0314
**Data/restraints/parameters**	2615/0/235	3261/0/184	2778/0/184	3008/1/192
**GooF on F^2^**	1.039	1.018	1.070	1.074
**Final R indices [I > 2σ** **(I)]**	R1 = 0.0410, wR2 = 0.0912	R1 = 0.0585, wR2 = 0.1320	R1 = 0.0356, wR2 = 0.0743	R1 = 0.0347, wR2 = 0.0756
**R indices (all data)**	R1 = 0.0578, wR2 = 0.0998	R1 = 0.1012, wR2 = 0.1582	R1 = 0.0429, wR2 = 0.0781	R1 = 0.0399, wR2 = 0.0787
**Largest diff. peak/hole, e Å^−3^**	0.255/−0.157	0.370/−0.236	0.171/−0.153	0.245/−0.139
**CCDC number**	2153868	2153866	2153867	2153869

## Data Availability

Not applicable.

## Supplementary Materials

The following supporting information can be downloaded at: https://www.mdpi.com/article/10.3390/molecules27082479/s1, Table S1: Interatomic distances and selected bond angles.

## Sample Availability

Samples of the compounds **1**–**4** are available from the authors.
