# Supplementary material for: Experimental and Computational Structural Studies of 2,3,5-Trisubstituted and 1,2,3,5-Tetrasubstituted Indoles as Non-Competitive Antagonists of GluK1/GluK2 Receptors
Source: Molecules. 2022 Apr 12;27(8):2479. doi: 10.3390/molecules27082479 (PMC9032324; doi:10.3390/molecules27082479)
Supplement: Supplementary file 1 [file molecules-27-02479-s001.zip › molecules-1660322-supplementary.pdf]

Supplementary Information

Experimental and computational structural studies of 2,3,5-trisubstituted and 1,2,3,5-tetrasubstituted indoles as non-competitive antagonists of GluK1/GluK2 receptors

Agata Bartyzel [1]*, Agnieszka A. Kaczor [2,3]*, Ghodrat Mahmoudi [4], Ardavan Masoudiasl [4], Tomasz M. Wróbel [2], Monika Pitucha [5] and Dariusz Matosiuk [2]

[1] Department of General and Coordination Chemistry and Crystallography, , Institute of Chemical Sciences, Faculty of Chemistry, Maria Curie-Skłodowska University, M. Curie-Skłodowskiej Sq. 2, PL-20031 Lublin, Poland; agata.bartyzel@mail.umcs.pl

[2] Department of Synthesis and Chemical Technology of Pharmaceutical Substances with Computer Modeling Laboratory, Faculty of Pharmacy, Medical University of Lublin, 4A Chodźki St., PL-20093 Lublin, Poland; agnieszka.kaczor@umlub.pl, tomasz.wrobel@umlub.pl, dariusz.matosiuk@umlub.pl

[3] School of Pharmacy, University of Eastern Finland, Yliopistonranta 1, P.O. Box 1627, FI-70211 Kuopio, Finland

[4] Department of Chemistry, Faculty of Science, University of Maragheh, P.O. Box 55181-83111, Maragheh, Iran, ghodratmahmoudi@maragheh.ac.ir, ardavan.masoudiasl@gmail.com

[5] Independent Radiopharmacy Unit, Faculty of Pharmacy with Division of Medical Analytics, Medical University of Lublin, 4A Chodźki St., PL-20093 Lublin, Poland, monika.pitucha@umlub.pl

* Correspondence: agata.bartyzel@mail.umcs.pl, tel. +48 81 537 7998, agnieszka.kaczor@umlub.pl, tel. +48 81 448 7273

Supplementary Table

Table S1. Interatomic distances and selected bond angles.

Bond lengths (Å)			
**1**			
C(1)-C(2)	1.376(2)	C(11)-C(12)	1.384(3)
C(1)-N(1)	1.386(2)	C(11)-C(17)	1.399(3)
C(1)-C(11)	1.470(2)	C(11)-C(12a)	1.451(3)
C(2)-C(4)	1.431(2)	C(12)-C(13)	1.381(4)
C(2)-C(3)	1.493(2)	C(13)-C(14)	1.358(3)
C(4)-C(10)	1.399(2)	C(12a)-C(13a)	1.379(4)
C(4)-C(5)	1.410(2)	C(13A)-C(14)	1.411(3)
C(5)-C(6)	1.375(2)	C(14)-C(16a)	1.363(3)
C(6)-O(1)	1.375(2)	C(14)-O(2)	1.367(2)
C(6)-C(8)	1.401(2)	C(14)-C(16)	1.423(3)
C(7)-O(1)	1.422(2)	C(15)-O(2)	1.425(3)
C(8)-C(9)	1.377(2)	C(16)-C(17)	1.387(4)
C(9)-C(10)	1.392(2)	C(15a)-O(2)	1.386(3)
C(10)-N(1)	1.376 (2)	C(16a)-C(17a)	1.395(4)
C(11)-C(17a)	1.348(3)		

**2**			
C(1)-C(2)	1.367(3)	C(9)-N(1)	1.380(2)
C(1)-N(1)	1.395(2)	C(10)-N(1)	1.461(3)
C(1)-C(12)	1.476(3)	C(10)-C(11)	1.489(4)
C(2)-C(4)	1.426(3)	C(12)-C(13)	1.388(3)
C(2)-C(3)	1.499(3)	C(12)-C(18)	1.401(3)
C(4)-C(5)	1.399(3)	C(13)-C(14)	1.380(3)
C(4)-C(9)	1.408(3)	C(14)-C(15)	1.377(3)
C(5)-C(6)	1.375(3)	C(15)-O(1)	1.370(2)
C(6)-C(7)	1.387(3)	C(15)-C(17)	1.388(3)
C(7)-C(8)	1.376(3)	C(16)-O(1)	1.412(3)
C(8)-C(9)	1.392(3)	C(17)-C(18)	1.370(3)
**3**			
C(1)-C(2)	1.371(3)	C(8)-C(9)	1.371(3)
C(1)-N(1)	1.394(3)	C(9)-C(10)	1.399(3)
C(1)-C(13)	1.484(3)	C(10)-N(1)	1.381(3)
C(2)-C(4)	1.433(3)	C(11)-N(1)	1.457(3)
C(2)-C(3)	1.497(3)	C(11)-C(12)	1.520(3)
C(4)-C(10)	1.406(3)	C(13)-C(14)	1.394(3)
C(4)-C(5)	1.409(3)	C(13)-C(18)	1.395(3)
C(5)-C(6)	1.383(3)	C(14)-C(15)	1.380(3)
C(6)-O(1)	1.381(2)	C(15)-C(16)	1.384(3)
C(6)-C(8)	1.412(3)	C(16)-C(17)	1.378(3)
C(7)-O(1)	1.420(2)	C(17)-C(18)	1.386(3)
**4**			
C(1)-C(10)	1.381(3)	C(10)-C(11)	1.416(3)
C(1)-N(1)	1.390(3)	C(11)-C(17)	1.411(3)
C(1)-C(2)	1.468(3)	C(11)-C(12)	1.411(3)
C(2)-C(3)	1.394(3)	C(12)-C(13)	1.380(3)
C(2)-C(7)	1.422(3)	C(13)-O(1)	1.376(3)
C(3)-C(4)	1.385(3)	C(13)-C(15)	1.405(3)
C(4)-C(5)	1.384(3)	C(14)-O(1)	1.422(3)
C(5)-C(6)	1.384(3)	C(15)-C(16)	1.377(3)
C(6)-C(7)	1.390(3)	C(16)-C(17)	1.396(3)
C(7)-C(8)	1.510(3)	C(17)-N(1)	1.386(2)
C(8)-C(9)	1.515(3)	C(18)-N(1)	1.457(3)
C(9)-C(10)	1.491(3)	C(18)-C(19)	1.515(3)
Bond angles (º)			
**1**			
C(14)-O(2)-C(15A)	119.5(2)	C(6)-O(1)-C(7)	117.0(1)
C(14)-O(2)-C(15)	118.1(2)		
**2**			
C(15)-O(1)-C(16)	118.2(2)	N(1)-C(10)-C(11)	112.8(2)
**3**			

C(6)-O(1)-C(7)	116.5(2)	N(1)-C(11)-C(12)	113.1(2)
**4**			
C(13)-O(1)-C(14)	115.6(2)	N(1)-C(18)-C(19)	112.8(2)
Torsion angles (º)			
**1**			
C(5)-C(4)-C(10)-C(9)	0.1(2)	C(13A)-C(14)-O(2)-C(15A)	178.4(2)
C(2)-C(4)-C(10)-N(1)	1.0(2)	C(13)-C(14)-O(2)-C(15)	170.9(2)
C(2)-C(1)-C(11)-C(12)	37.3(3)	C(16)-C(14)-O(2)-C(15)	-9.0(3)
C(2)-C(1)-C(11)-C(12A)	-44.8(2)	N(1)-C(1)-C(11)-C(17A)	-44.3(2)
C(2)-C(1)-C(11)-C(17)	-147.1(2)	N(1)-C(1)-C(11)-C(12)	-142.1(2)
C(2)-C(1)-C(11)-C(17A)	135.1(2)	N(1)-C(1)-C(11)-C(17)	33.5(2)
C(16A)-C(14)-O(2)-C(15A)	3.2(3)	N(1)-C(1)-C(11)-C(12A)	135.8(2)
**2**			
C(2)-C(4)-C(9)-N(1)	-0.9(2)	N(1)-C(1)-C(12)-C(18)	61.2(3)
C(5)-C(4)-C(9)-C(8)	-1.9(3)	C(11)-C(10)-N(1)-C(9)	77.4(3)
C(2)-C(1)-C(12)-C(13)	59.0(3)	C(11)-C(10)-N(1)-C(1)	-109.2(2)
N(1)-C(1)-C(12)-C(13)	-122.5(2)	C(14)-C(15)-O(1)-C(16)	7.4(3)
C(2)-C(1)-C(12)-C(18)	-117.3(2)	C(17)-C(15)-O(1)-C(16)	-172.0(2)
**3**			
C(2)-C(4)-C(10)-N(1)	-2.2(2)	N(1)-C(1)-C(13)-C(18)	-117.4(2)
C(5)-C(4)-C(10)-C(9)	-2.4(3)	C(12)-C(11)-N(1)-C(10)	-82.2(2)
C(2)-C(1)-C(13)-C(14)	-112.2(2)	C(12)-C(11)-N(1)-C(1)	80.7(2)
N(1)-C(1)-C(13)-C(14)	64.6(3)	C(5)-C(6)-O(1)-C(7)	5.7(3)
C(2)-C(1)-C(13)-C(18)	65.8(3)	C(8)-C(6)-O(1)-C(7)	-175.5(2)
**4**			
N(1)-C(1)-C(2)-C(3)	-13.7(3)	C(12)-C(11)-C(17)-C(16)	0.0(3)
C(3)-C(2)-C(7)-C(6)	0.4(3)	C(19)-C(18)-N(1)-C(17)	-91.9(2)
C(1)-C(2)-C(7)-C(8)	-2.3(3)	C(19)-C(18)-N(1)-C(1)	89.1(2)
C(2)-C(1)-C(10)-C(9)	-3.3(3)	C(12)-C(13)-O(1)-C(14)	-1.8(3)
C(10)-C(11)-C(17)-N(1)	-0.1(2)	C(15)-C(13)-O(1)-C(14)	178.9(2)
